# Update on the application of mesenchymal stem cell-derived exosomes in the treatment of Parkinson's disease: A systematic review

**DOI:** 10.3389/fneur.2022.950715

**Published:** 2022-10-03

**Authors:** Shu-fen Liu, Lin-yi Li, Jian-long Zhuang, Mi-mi Li, Li-chao Ye, Xiang-rong Chen, Shu Lin, Chun-nuan Chen

**Affiliations:** ^1^Department of Neurology, The Second Affiliated Hospital, The Second Clinical Medical College, Fujian Medical University, Quanzhou, China; ^2^Prenatal Diagnosis Center, Quanzhou Women's and Children's Hospital, Quanzhou, China; ^3^Department of Neurosurgery, The Second Affiliated Hospital, The Second Clinical Medical College, Fujian Medical University, Quanzhou, China; ^4^Centre of Neurological and Metabolic Research, The Second Affiliated Hospital of Fujian Medical University, Quanzhou, China; ^5^Diabetes and Metabolism Division, Garvan Institute of Medical Research, Darlinghurst, NSW, Australia

**Keywords:** mesenchymal stem cells, exosomes, Parkinson's disease, treatment, miRNA

## Abstract

Parkinson's disease (PD) has become the second largest neurodegenerative disease after Alzheimer's disease, and its incidence is increasing year by year. Traditional dopamine replacement therapy and deep brain stimulation can only alleviate the clinical symptoms of patients with PD but cannot cure the disease. In recent years, stem cell therapy has been used to treat neurodegenerative diseases. Many studies have shown that stem cell transplantation has a therapeutic effect on PD. Here, we review recent studies indicating that exosomes derived from mesenchymal stem cells also have the potential to treat PD in animal models, but the exact mechanism remains unclear. This article reviews the mechanisms through which exosomes are involved in intercellular information exchange, promote neuroprotection and freely cross the blood-brain barrier in the treatment of PD. The increase in the incidence of PD and the decline in the quality of life of patients with advanced PD have placed a heavy burden on patients, families and society. Therefore, innovative therapies for PD are urgently needed. Herein, we discuss the mechanisms underlying the effects of exosomes in PD, to provide new insights into the treatment of PD. The main purpose of this article is to explore the therapeutic potential of exosomes derived from mesenchymal stem cells and future research directions for this degenerative disease.

## Introduction

Parkinson's disease (PD) mainly affects older people, with a prevalence of 1–2% (1, 2). Patients with PD have clinical manifestations of motor symptoms, such as bradykinesia, resting tremor, postural and gait disorders, as well as non-motor symptoms, such as depression, sleep disorders, dementia and peripheral injuries. The onset of PD is very difficult to detect, and patients with PD are often not identified in early stages. The early symptoms of PD are atypical, and can easily be missed or misdiagnosed. Moreover, patients with advanced PD cannot care for themselves, and their quality of life is severely affected, thus posing a heavy burden on patients and their families (3).

Currently, various methods are used to treat PD, most commonly medication and surgery. Drugs such as pharmacological formulations of dopamine substitutes are beneficial in early stages of the disease, but long-term use decreases efficacy and can even cause serious adverse reactions (4). Deep brain stimulation (DBS), another treatment for PD, is increasingly used worldwide. Studies have shown that music, dancing and physical exercise can both improve the symptoms of patients with PD and decrease PD risk (5, 6). However, these treatment methods only relieve the symptoms of PD but cannot completely prevent the degeneration process. Therefore, innovative therapies, such as inducing neuroprotection of surviving dopamine neurons and reestablishing dopamine balance, are urgently needed.

The pathological features of PD are mainly characterized by dopaminergic neuron loss in the substantia nigra (SN) (7). Consequently, cell replacement therapy may be a straightforward and potentially curative treatment. Mesenchymal stem cells (MSCs) are a type of stem cells with multiple differentiation potential. Under certain conditions, MSCs can differentiate into neurons. Because of their self-differentiation ability, and the paracrine effects of secretion of growth factors and cytokines, MSCs may have therapeutic potential (8, 9). Exosomes, an important paracrine factor of MSCs, have also been reported to be a potential treatment for central nervous system diseases.

Therefore, the purpose of this review is to explore the therapeutic potential of exosomes derived from MSCs in PD and their future development prospects for treating degenerative diseases.

## Pathophysiology of PD

### The etiology of PD

The etiology of PD remains unclear but is believed to involve the interaction of aging, genetics and environmental factors. The pathology is associated with a decrease in dopaminergic neurons, a decrease in neurons in the SN and other brain structures, and the formation of Lewy bodies. The diagnosis of PD is mainly based on medical history and clinical manifestations, and no specific diagnostic test is available. Auxiliary imaging studies such as positron emission tomography and magnetic resonance imaging are helpful for the clinical diagnosis of PD (1, 2). Genes are an important factor in PD. Studies have indicated that the prevalence of PD among the first-degree relatives of patients with PD is two to three times higher than that in the general population (10). The primary risk factor among external factors is aging.

The incidence of PD increases exponentially with age. Environmental exposure is another risk factor. A meta-analysis has shown that exposure to pesticides, head injury, rural living, taking beta-blockers, engaging in agriculture activities and drinking well water can increase the risk of PD, whereas alcohol consumption, smoking, drinking coffee, and using non-steroidal anti-inflammatory drugs or calcium channel blockers can decrease the risk of PD (11). A study has also shown that twins with PD tend to smoke less than twins without PD (12). Therefore, people who smoke may have lower risk of PD. In short, PD is generally believed to be caused by a variety of factors.

### The genetics of PD

A series of major gene mutations are known to cause autosomal dominant and recessive PD. For example, α-Synuclein (SNCA), parkin 2 (PARK2), PTEN-induced putative kinase 1 (PINK1), park7, Leucine-rich repeat kinase 2 (LRRK2), bone marrow stromal cell antigen 1 (BST1), and microtubule-associated protein tau (MAPT) may cause familial PD. Other genetic defects at other loci may be susceptibility sites for sporadic PD (13). Mutations in the LRRK2 and Parkin genes are considered the most common causes of dominant and recessive PD, respectively (14).

Previous studies have shown that mutations in the glycocerebral glycosidase 1 (GBA1) gene are the most susceptible risk factor for PD. In addition, the GBA1 mutation rate in patients with PD is higher than that in healthy people (15). GBA1 gene mutation leads to abnormal folding of glucocerebrosidase (GCase), thus affecting the endoplasmic reticulum (ER), lysosomes and mitochondria (13). Abnormally folded glucocerebrosidase in the ER enhances the ubiquitin-proteasome system and ER stress. The ER stress in turn triggers the unfolded protein response as well as ER degradation, thereby increasing cell apoptosis. The presence of misfolded GCase in lysosomes, coupled with a decrease in wild-type GCase levels, results in SNCA degradation through delayed chaperone-mediated autophagy, and leads to the accumulation and aggregation of SNCA. Simultaneously, lysosome dysfunction leads to decreased autophagosome clearance and to the accumulation of cell debris. GBA1 mutation promotes the formation of free radicals, and decreases the production of ATP, oxygen consumption and the membrane potential (15), thereby perturbing normal mitochondrial function. In addition, GBA1 heterozygous mutation alters the lysosomal enzyme that converts glycosylceramide to ceramide and also increases the risk of PD (16).

### Lewy bodies

The pathological features of PD are progressive loss of dopaminergic neurons in the substantia nigra pars compacta and the deposition of Lewy bodies (13). Both pathological features of PD extensively involve other parts of the central nervous system and surrounding tissues (17–19). Oral administration of levodopa, the precursor of dopamine, can relieve most of the symptoms. Lewy bodies, a characteristic marker of PD brain tissue, are composed of misfolded neuron inclusions formed by aggregates of SNCA. Lewy bodies are composed of a dense core in the center, surrounded by fine filamentous fainting. The formation of Lewy bodies may be associated with mitochondrial defects (20). The SNCA gene, located on chromosome 4q21, encodes a protein that plays an important role in the pathophysiology of PD and was the first PD autosomal dominant gene discovered (21). Animal model studies and human cadaver studies have shown clear cellular and molecular changes in PD brain tissues, such as subtle changes in specific gene expression, neuroinflammation, oxidative stress, mitochondrial dysfunction, apoptosis, autophagy and glial cell-derived neurotrophic factor deficiency. Imbalances in non-coding RNAs and effects of genomic variation are also associated with the pathogenesis of PD (22). Furthermore, the interactions between glial cells and miRNAs play crucial roles in the occurrence and development of disease (22, 23).

### Treatment of PD

The clinical manifestations of PD can be divided into motor symptoms and non-motor symptoms. Both can affect patients' ability to work and conduct daily life activities. Therefore, drug treatment aims to improve symptoms, avoid or decrease adverse reactions, and improve work ability and quality of life. Early diagnosis and treatment are advocated. Currently, the treatments for PD include pharmacological and non-pharmacological treatments. The former mainly consists of levodopa preparations, dopamine agonists, monoamine oxidase-B (MAO-B) inhibitors, anticholinergic drugs and amantadine. The latter mainly includes DBS and exercise therapy (24). As the availability of treatment methods increases, the management of patients with PD must be individualized (25).

#### Drug therapy

Levodopa, the precursor of dopamine, is the most commonly used drug in clinical practice. It can further supplement the loss of dopamine and can improve patients' daily lives and movement; however, it cannot improve mental function. Dopamine agonists alleviate the symptoms of PD by enhancing the activity of dopamine receptors, but they may cause abnormal reward regulation-related behaviors, nausea, orthostatic hypotension, dizziness, drowsiness, hallucinations and edema. MAO-B inhibits monoamine oxidase, thereby inhibiting the degradation of dopamine into dihydroxyphenylacetic acid and prolonging the effect of dopamine. It is often used as an adjuvant drug for patients with a weakened levodopa response. Anticholinergic drugs function by blocking postsynaptic acetylcholine receptors. Adverse effects of anticholinergic medications may include mental confusion, mydriasis with decreased vision, dry eyes, dry mouth, constipation and urinary retention. These drugs are contraindicated for patients with acute glaucoma and should be used for patients with peptic ulcers. The pharmacological mechanism of amantadine is unknown; however, thus drug can induce edema and hallucination (26). Pharmacotherapy can improve patients' symptoms to some extent but cannot change the progressive course of PD.

#### DBS

In 2002, DBS was approved by the US Food and Drug Administration for the treatment of PD and is commonly used in advanced PD. This surgery enables persistent microcurrent stimulation of specific sites in the deep brain *via* electrodes implanted into the brain, to eliminate or inhibit potential pathological neural activity. Two common targets are the subthalamic nucleus and the globus pallidus interna. This surgery can effectively improve the symptoms of patients with PD, such as bradykinesia, tremor, rigidity, and conversion pulsation. However, whether it is effective in the treatment of gait disorders, speech and cognitive dysfunction has not been determined. Cerebral hemorrhage venous thrombosis, phlebitis, pneumonia, urinary tract infections and pulmonary embolism are the common complications (27, 28). As with drug therapy, DBS can neither completely treat PD nor prevent the progression of the neurodegenerative process of PD (29).

#### The roles of MSCs in PD treatment

MSCs have a high capacity for self-propagation and can differentiate into neural precursor cells. Therefore, these cells are considered an ideal source to replace lost cells in degenerative diseases such as PD (30). Early studies have shown that the expression of dopaminergic markers such as tyrosine hydroxylase (TH), dopamine transporter (DAT) and dopamine D2 receptor in the substantia nigra pars compacta and the functional release of dopamine in the striatum are greater in PD rats than controls. Moreover, PD rats showed behavioral improvements. Therefore, researchers have hypothesized that MSC transplantation might prevent the progressive death of neurons and subsequently restore the function of dopamine neurons, thus leading to improvement in early behavior (31). However, few clinical studies have examined the treatment of PD with MSCs. In a study of seven patients with PD, motor function has been found to improve after injection of MSCs (32). Overall, after transplantation of MSCs, the course of PD could be modified, thereby ameliorating both motor and non-motor symptoms of PD (33, 34).

## MSCs for PD

### MSCs

MSCs are stem cells with self-renewal ability and multiple differentiation potential. The standards for MSCs established by the International Society for Cell Therapy are as follows: MSCs are stem cells ① that adhere to plastic ②have the potential to differentiate into osteoblasts, chondrocytes and adipocytes ③ and are characterized by CD105, CD73, and CD90 positivity, and CD45, CD34, CD14 CD11b, CD19, and human leukocyte antigen-DR (HLA-DR) negativity (35, 36).

#### The origin of MSCs

**Figure 1:**
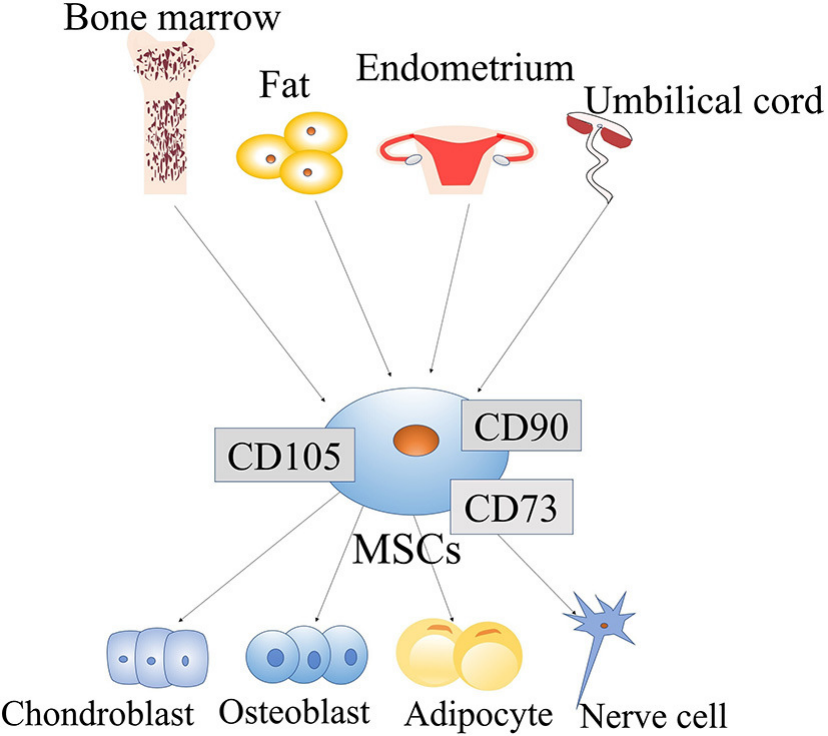
Origin and differentiation of mesenchymal stem cells (MSCs): MSCs come from a wide range of sources, such as the bone marrow, fat, endometrium, and umbilical cord. MSCs have the potential for self-renewal and multi-lineage differentiation. They can differentiate into various lineage of tissues, include chondroblasts, osteoblasts, adipocytes, and neuronal cells. MSCs are easily obtainable from a wide range of sources, and their ability to differentiate multi-directionally allows them to be applied in a variety of fields.

MSCs have many sources and can be extracted from bone marrow, umbilical cord blood and adipocytes (37, 38). Many studies have shown that MSCs can also be extracted from the endometrium and menstrual blood (39) (Figure 1). Moreover, MSCs have been demonstrated to be useful in the treatment of many diseases, such as acute kidney injury (40), and wounds (41). MSCs are useful for not only PD but also other diseases. Therefore, MSCs are a promising treatment and have been more widely studied in recent years.

#### Differentiation of MSCs

Increasing evidence indicates that MSCs can transdifferentiate into certain cell lineages. In adults, MSCs are generally considered to have mesodermal origin (42) and therefore can differentiate into mesodermal lineages, such as myocytes, chondrocytes, osteocytes, adipocytes, tenocytes and stromal fibroblasts. They are also able to differentiate into endodermal and ectodermal lineages, such as hepatocytes, neurons and glia (43). Epigenetic changes can mediate the MSC phenotypic transition. For example, exposure of bone marrow stromal cells (BMSCs) to epigenetic modulators and neural induction mediators can lead to their neuronal differentiation. Under the action of adiposity-inducing factor, the morphology of these cells undergoes similar changes (44). Therefore, epigenetic regulators can regulate the differentiation potential of MSCs into multiple cell types (Figure 1).

### Current research findings on MSCs in PD treatment

In recent years, researchers have increasingly paid attention to PD. Recent research has shown that in a mouse model of PD, knockdown of the RNA binding protein polypyrimidine tract-binding protein (PTB) in astrocytes can directly transform them into functional neurons and consequently effectively improve PD-related dyskinesia. More importantly, the use of antisense oligonucleotides that inhibit PTB has achieved similar therapeutic effects (45), thus providing a major breakthrough in the treatment of PD. Furthermore, various studies have shown that MSCs have potential in the treatment of PD. New evidence showing that stem cell-based cell transplantation instead of dysfunctional dopaminergic neurons in the treatment of PD has attracted widespread attention (46). In that study, transplantation of MSCs into PD animal model increased dopaminergic neurons in the animal brain and led to improvements in behavior; however, whether MSCs can be directly transformed into dopaminergic neurons remains to be confirmed (46). Another study has transplanted green fluorescent protein–labeled MSCs into a PD rat model and shown significant improvements in animal behavior; moreover, green fluorescent protein–labeled tyrosine hydroxylase positive cells were found in the brain in the rat model. These results suggest that MSCs are likely to replace missing dopaminergic neurons and have potential in the treatment of PD (47).

Early application of drug therapy for PD can effectively improve motor symptoms in patients. DBS is a surgical operation aimed at improving the motor symptoms and quality of life of patients with PD (48). However, both treatments can only improve short-term symptoms, whereas long-term use may have some adverse effects (49). Because current treatment methods cannot alter the underlying pathology, they cannot cure PD. The purpose of MSC therapy is to replace degenerative dopaminergic neurons, thus targeting the pathogenesis of PD. Autologous MSC transplantation is harmless to recipients. MSC transplantation can regenerate and recover nerves in damaged tissue, thus bringing hope for the treatment of PD (30). Current experimental evidence shows that MSCs derived from bone marrow, fat and cord blood significantly improve the symptoms of PD (50).

### The mechanism through which MSCs play a beneficial role in PD

#### The mechanism of MSCs in the treatment of PD

The mechanism of MSCs in the treatment of PD has not been well-elucidated. The current research findings are as follows. First, MSCs secrete neurotrophic growth factors, including glial cell-derived neurotrophic factor (GDNF), vascular endothelial growth factor and brain-derived neurotrophic factor (51, 52), which are further secreted under specific culture conditions (53). Neurotrophic growth factor is necessary for the growth and development of neurons. Some neurotrophic factors support cell survival and protect against neuronal degeneration (54). In many preclinical models of neurological diseases, including amyotrophic lateral sclerosis, PD, and multiple system atrophy transgenic animal models, neurotrophic factors have been found to increase the survival rate of neurons (23, 55). The same mechanism is also believed to be involved in the treatment of PD. Second, MSCs are weak in immunogenicity and exert immunosuppressive effects through various mechanisms, such as inhibiting T cells and B cells, dendritic cells, natural killer cells and neutrophils (3). Therefore, they may confer potential benefits in the treatment of allograft rejection and suppression of autoimmune diseases. Moreover, MSCs have anti-apoptosis and anti-inflammatory effects, and can improve the prognosis of PD. They also protect against SNCA-induced degeneration of dopaminergic neurons, as confirmed in a neurotoxin-induced PD model (56, 57). In recent years, studies have attributed the therapeutic effects of MSCs to paracrine effectors. Notably, the isolation process of MSCs is simple, and the cells have high expansion capacity, high biological safety, low ethical challenges and low tumorigenic risk (58–60). In summary, the benefits of MSCs in treating PD include their availability, low immunogenicity multiple differentiation capability, and secretion of neurotrophic growth factors and paracrine factors.

#### Clinical trials

Currently, MSCs in the treatment of PD have been examined in 12 clinical trials. Five clinical trials are in the recruitment stage, and two clinical trials are no longer available (Table 1). The two discontinued trials involved eight or 12 injections with cord blood MSCs; patients were followed up to observe changes in activities of daily living and quality of life, to evaluate the efficacy of the drugs. The reasons for discontinuation remain unknown. In the five clinical trials in recruitment, MSCs and placebo experiments, autologous MSCs and allogeneic MSC transplantation experiments have been performed to compare their efficacy. However, no results have been reported. The number of clinical trials is small, and therefore the application of MSCs in the treatment of PD requires further research.

**Table 1:** Clinical trials using MSCs in the treatment of PD.

	**Status**	**Study title**	**Conditions**	**Interventions**
1	Enrolling by invitation	Umbilical Cord Derived MSCs^a^ Therapy in PD^b^	PD	Biological: MSC
2	Unknown	MSCs Transplantation to Patients With PD	PD	Biological: BM-MSCs^c^
3	Completed	Allogeneic BM-MSCs Therapy for Idiopathic PD	PD	• Biological: Allogeneic BM-MSCs (1 × 10^6^ MSC/kg) • Biological: Allogeneic BM-MSCs (3 × 10^6^ MSC/kg) • Biological: Allogeneic BM-MSCs (6 × 10^6^ MSC/kg • Biological: Allogeneic BM-MSCs (10 × 10^6^ MSC/kg)
4	No longer available	Individual Patient Expanded Access IND of Hope Biosciences Autologous Adipose-derived Mesenchymal Stem Cells for PD	PD	Drug: HB-adMSCs^d^
5	Recruiting	Phase IIa Randomized Placebo Controlled Trial: Mesenchymal Stem Cells as a Disease-modifying Therapy for iPD^e^	PD	• Drug: MSC+Placebo • Drug: MSC • Drug: Placebo
6	Active, not recruiting	Use of MSCs Differentiated into NSCs^f^ in People with PD	PD	Biological: Umbilical cord derived MSCs
7	Terminated	Autologous Mesenchymal Stem Cell Transplant for Parkinson's Disease	PD	Procedure: Autologous BM-MSCs transplant
8	Recruiting	Parkinson's Disease Therapy Using Cell Technology	MSCs Transplantation	• Biological: Autologous MSCs • Other: Placebo
9	No longer available	HB-adMSCs for the Treatment of Parkinson's Disease	PD	Biological: HB-adMSCs^g^
10	Recruiting	Parkinson's Disease (Early and Moderate)	PD	• Biological: HB-adMSCs • Other: Placebo
11	Recruiting	Potential Use of Autologous and Allogeneic Mesenchym-al Stem Cells in Patients with Multiple System Atrophy	• Multiple system atrophy • Parkinsonism • Multiple system atrophy, Parkinson's variant	• Biological: Autologous adMSCs implantation • Biological: Allogeneic umbilical cord mesenchymal stem cell implantation • Biological: Allogeneic umbilical cord mesenchymal stem cell and adipose secretome implantation
12	Recruiting	Clinical Trial for Parkinson's Disease Using Allogeneic HB-adMSCs (Early and Moderate) (PD)	PD	• Biological: Biological/Vaccine: Allogeneic HB-adMSCs • Other: Placebo

Five clinical trials are recruiting, and two clinical trials have been discontinued. MSCs in the treatment of PD have been examined in only 12 clinical trials. Further development is therefore necessary. An additional movie file shows more details.

^a^MSCs, mesenchymal stem cells.

^b^PD, Parkinson's Disease.

^c^BM-MSCs, Bone marrow derived MSC.

^d^adMSCs, Adipose-derived Mesenchymal Stem Cells.

^e^iPD, Idiopathic Parkinson's Disease.

^f^NSCs, Neural Stem Cells.

^g^HB-adMSCs, Hope Biosciences autologous adipose derived mesenchymal stem cells.

#### Adverse effects of mesenchymal stem cells in PD

MSCs have been used to treat many diseases because of their differentiation potential, but several problems remain. For example, MSCs cannot effectively cross the blood-brain barrier without the help of osmotic agents. Moreover, MSC have only a low survival rate in hosts after transplantation (61). In addition, allogeneic MSCs can cause transplant rejection, thus causing harm to patients. Therefore, many researchers have used different approaches to solve these problems.

Researchers have found that exosomes derived from MSCs can freely cross the blood-brain barrier, thus decreasing transplant rejection and effectively ameliorating PD symptoms. Therefore, exosomes may be a better treatment for PD with fewer side effects.

## MSC derived exosomes in PD

### Application of MSC derived exosomes in PD

In recent years, with the development of MSCs in the treatment of PD, many researchers have attributed the positive effects of MSCs in the treatment of PD to exosomes. Exosomes are extracellular vesicles released by MSCs. MSCs are regulated by the extracellular vesicles (EVs) that they secrete (50, 51). EVs are involved in the exchange of materials between cells (62).

EVs are released by a variety of eukaryotic cells, and contain many substances including RNA, DNA and major nutrients. EVs play important roles in intercellular communication (63). In recent years, EVs have gradually been applied in tumor prevention. EVs are selectively delivered to target organs/tumors after loading with therapeutic agents (e.g., drugs). Cell-free therapy is another treatment option. However, EVs have several limitations. For example, tumor-derived EVs promote angiogenesis and consequently promote tumor growth, and also play a role in tumor development (64–66). In addition, the expression of exosome related proteins and genes increases after metformin treatment of glioblastoma multiforme (GBM) cells, and the growth of cancer cells is inhibited. Thus, activation of the exosome secretion pathway plays an important role in cancer treatment (67). Feghhi et al. have found that exosomes promote angiogenesis (68).

According to differences in size, density, surface markers and sources, EVs can be further subdivided into apoptotic bodies, cellular microparticles and exosomes (69). Apoptotic bodies are the EVs with the largest diameter formed by MSCs in apoptosis. Cellular microparticles are formed by budding of the MSC cell membrane (70). Exosomes, the most typical EVs, were first discovered in mature sheep reticulocytes in 1983 (35). Exosomes are a subtype of vesicles that can be released by various cells in all living systems (52, 53). In addition, studies increasingly indicate that exosomes derived from MSCs can effectively improve PD symptoms. Experimental models of PD treated with exosomes secreted by MSCs have shown that exosome treatment can avoid the adverse effects caused by MSC transplantation alone, such as poor differentiation (71). Moreover, studies have shown that injection of exosomes secreted by MSCs into the substantia nigra and striatum partially reverse motor and histological symptoms in a 6-OHDA PD rat model (72). In another study in a 6-OHDA rat model of PD, injection of human bone marrow stromal cells (hBMSC) derived exosomes, compared with hBMSC self-transplantation, has been found to be more effective in rescuing dopaminergic neurons and inducing higher levels of neuronal differentiation (73) (Table 2). MSC derived exosomes can transfer miRNAs to neuronal cells. MiR-133b is expressed in midbrain dopaminergic neurons and is a type of miRNA that regulates the production of tyrosine hydroxylase. Exosomes with high miR-133b content can promote the growth of neurites (74). Researchers have discovered that patients with PD lack miR-133b (75). Exosomes that are released by genetically modified macrophages contain antioxidants, catalase and GDNF, which can effectively improve the symptoms of PD (76–78). Exosomes derived from MSCs have been studied in a variety of PD models and have therapeutic potential in the treatment of PD. Therefore, exosomes are considered a promising treatment for PD.

**Table 2:** Application of mesenchymal stem cell (MSC) therapy in the treatment of PD.

**Disease**	**Treatment**	**Model**	**Results**	**References**
PD	Inject the hMSCs^a^ exosomes	Rat	• Motor coordination improved • The number of TH^b^-positive cells observed in the SNpc^c^ added	(62)
PD	• 6-OHDA^d^ control group • Sham group (sterile saline) • hBMSCs transplants • hBMSCs exosomes injection	Rat	• Motor coordination and balance of the animals was improved upon hBMSCs cell transplantation or exosomes injection • The fine motor movements improved upon exosomes injection • Injection of hBMSCs exosomes protects against TH damage in SNpc and striatum	(63)

Both MSCs and exosomes improve motor coordination and the number of tyrosine hydroxylase (TH) positive cells in the substantia nigra pars compacta (SNpc). However, exosomes can improve the fine motor movement, but whether MSCs also do so remains unknown. An additional movie file shows more details.

^a^hMSCs, human mesenchymal stem cells.

^b^TH, Tyrosine hydroxylase.

^c^SNpc, substantia nigra pars compacta.

^d^6-OHDA, 6-hydroxydopamine.

### The concept and structure of exosomes

**Figure 2:**
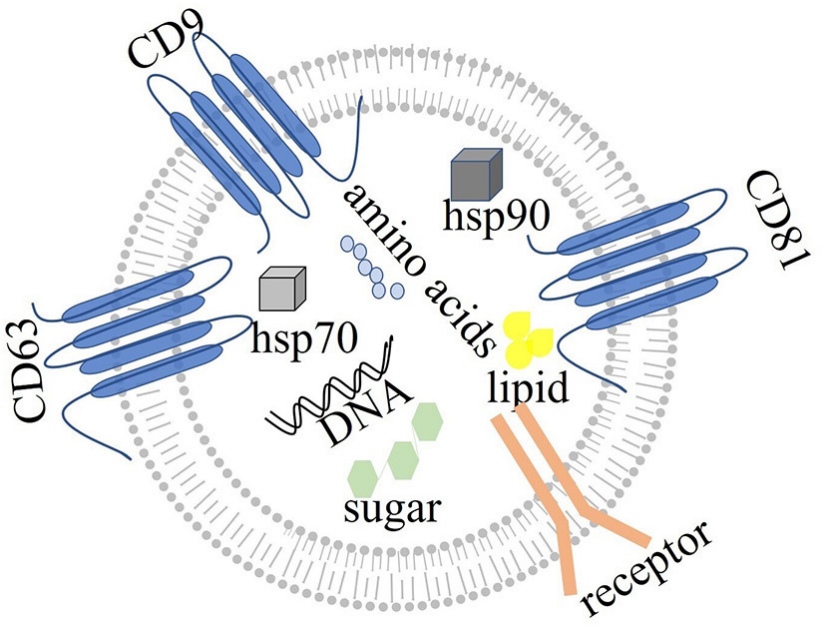
The structure of exosomes: exosomes, with single membranes, are rich in amino acids, sugar, lipids, and nucleic acids. Exosomes also express specific proteins, such as tetraspanin (CD63, CD81, and CD9), Heat Shock Protein 70 (hsp70), and Heat Shock Protein 90 (hsp90). Exosomes act by binding other cells through membrane receptors.

Exosomes are single membrane vesicles with the same topological structure as cells (79). The early endosomal membrane buds inward to form exosomal vesicles, which mature into multivesicular bodies, which in turn fuse with the cell membrane and are released into the extracellular space as exosomes (80–82). Multivesicular bodies may also degrade exosomes by binding lysosomes and circulating biomolecules to the plasma membrane (83). Both ESCRT-dependent and ESCRT independent mechanisms are involved in the production of exosomes. However, the interaction between these two mechanisms is unclear (84). The released exosomes exert their effects on receptor cells through endocytosis, ligand-receptor binding or direct binding (85, 86). Exosomes can be secreted by all cells in the body (87), such as adipocytes (88), and hypothalamic stem cells (89). Exosomes contain a variety of molecules, such as immune components, hormones, sugars, steroids, RNA, microRNAs, lipids and nucleic acid polymers (35, 90) (Figure 2). Exosomes can affect gene expression and protein biological activity *via* receptor cells through the messenger RNAs and proteins that they carry (91). For example, a study of colon cancer with KRAS gene mutation has shown that miR-10b is selectively increased in KRAS wild-type-derived exosomes, whereas miR-100 is increased in KRAS mutant-derived exosomes. This study has indicated that the KRAS gene may control the expression of miRNA by exosomes (92). Furthermore, exosomes from different sources carry different proteins and lipids, and many exosomes carry specific proteins. For example, myelin protein is specifically expressed in the exosomes secreted by oligodendrocytes (93). Some proteins, such as CD63, CD89, CD81, CD9 and CD82, and the heat shock proteins Heat Shock Protein 70 (HSP70) and Heat Shock Protein 90 (HSP90) (94), are specifically expressed in all exosomes and are called exosome-related-proteins, thereby providing a theoretical basis for the extraction and identification of exosomes. According to ExoCarta (Home-Exosome database), ~8,000 proteins and 194 lipids are known to be associated with exosomes (95). No evidence has indicated that exosomes affect gene expression related to neurodegenerative diseases. Moreover, exosomes from different sources may have different functions. For example, exosomes from antigen-presenting cells can express major histocompatibility complex class I and class II molecules on the cell surface, which help activate CD4+ and CD8+ T cells and induce specific immune responses. In another example, exosomes containing prostaglandins secreted by platelets have been suggested be involved in the inflammatory response (96, 97). As described above, exosomes are taken up by receptor cells through endocytosis, receptor ligand interaction or fusion with the cell membrane. These processes rely on the interactions between proteins on the surface of exosomes and their receptor cells. Exosomes containing Tetraspanin 8 (TSPAN8) and integrin α-4 have been shown to be easily detected by CD54+ pancreatic cells (98).

### Mechanisms through which exosomes derived from MSCs may have a beneficial effect on PD

**Figure 3:**
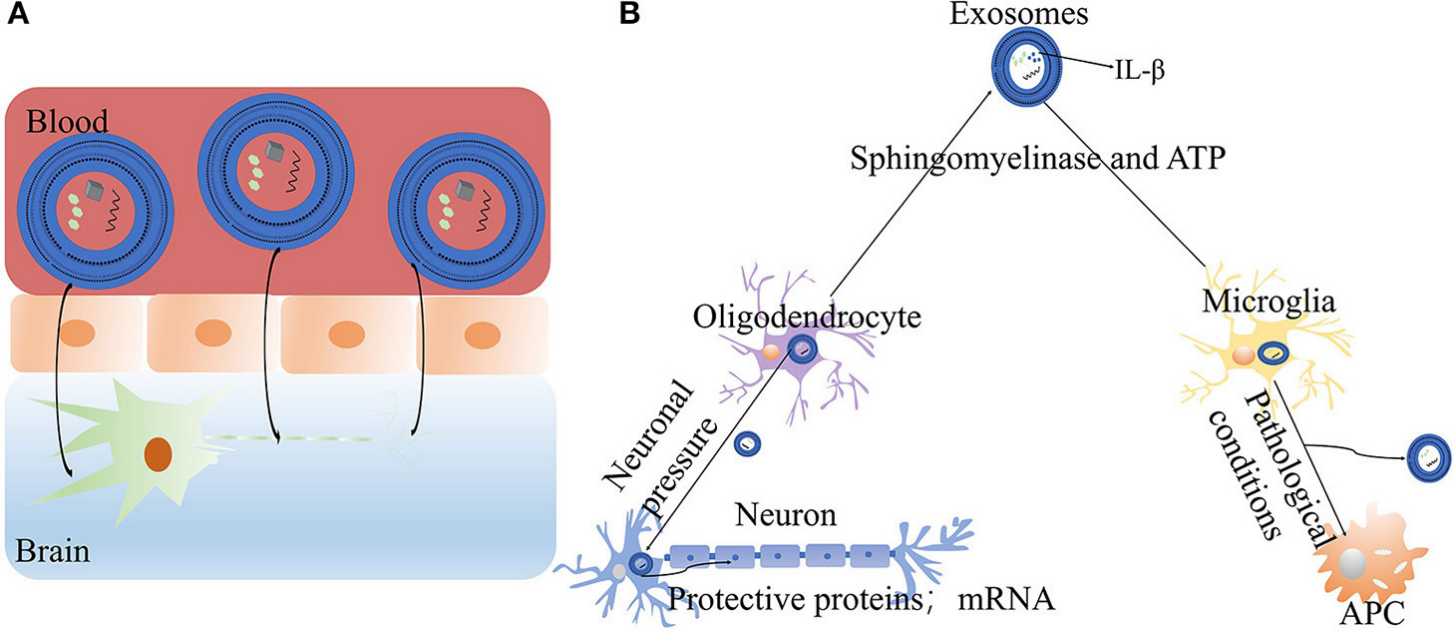
The mechanisms of exosomes in PD: **(A)** Exosomes can freely cross the blood-brain barrier, reach the central nervous system and exert therapeutic effects. **(B)** The roles of exosomes in neurons and cells: exosomes released by oligodendrocytes promote neuroprotection (ATP, adenosine-triphosphate; IL-β, Interleukin-1β).

According to clinical research reports, 98% of potentially effective drugs for the treatment of central nervous system diseases have failed in clinical trials because they cannot cross the blood-brain barrier (99). In general, the diameter of exosomes is only 30–150 nm (100, 101), which is sufficiently small to allow them to freely cross the blood-brain barrier and reach the central nervous system. In fact, some studies have demonstrated that exosomes have small diameters, low immunogenicity and long circulating half-lives, and consequently can be used as therapeutic signals or drug delivery carriers (102). For example, catalase is a promising treatment enzyme for PD, but the enzyme nanoparticles cannot cross the blood-brain barrier. In contrast, catalase administered by exosomes can reach the target neurons in a Parkinson's mouse model and accumulate in target cells (103). A recent study has discovered that in idiopathic PD, autophosphorylated ser (P)−1292 LRRK2 levels in urinary exosomes are elevated. Moreover, Ser (P)-1292 LRRK2 levels are higher in patients with PD with poorer cognition and are correlated with poor performance. Exosomes result in biochemical changes in LRRK2 in idiopathic PD (104). Exosomes can transfer protein, RNA and lipid components from one cell to another, and thus play an important role in intercellular communication. In addition, exosomes can communicate between cells under various physiological and pathological conditions, and therefore may play an important role in the treatment of PD (105–109). Frühbeis has reported that exosomes can mediate the interactions between neurons and oligodendrocytes. With the activation of neuronal pressure signal transduction, oligodendrocytes release exosomes, which are absorbed by neurons through endocytosis, and subsequently transfer protective proteins and mRNA to axons, thereby promoting neuroprotection (110, 111) (Figure 3). Furthermore, exosomes mediate neuron development, nerve regeneration and synaptic plasticity. Exosomes transfer regulatory elements to sites of nerve injury, thus aiding in protein synthesis and tissue regeneration (112). Some researchers have found that under pathological conditions, microglia are activated and transformed into antigen presenting cells by secreting exosomes. Under the stimulation with ATP and the activation of sphingomyelinase, the plasma membranes of microglia and astrocytes release the pro-inflammatory cytokine IL-β (113, 114). Therefore, exosomes may contribute to the antigen presentation of immune cells, participate in cell signal transduction, and have anti-inflammatory or pro-inflammatory characteristics (115, 116). A recent study has indicated that the motor symptoms and dopamine neurons in the substantia nigra striatum are upregulated after exosome treatment in PD mice, owing to the autophagy induced by exosomes (117).

However, anti-inflammatory drugs have been suggested to decrease the risk of PD, thus indicating that inflammation may promote the underlying PD process. Exosomes are believed to function in this way (118). All the above mechanisms may be involved in the treatment of PD by exosomes.

## Prospects of exosomes in the treatment of PD

In 1817, James Parkinson's described PD in an article entitled “Tremor Paralysis.” Since then, understanding of PD has made great progress (119, 120). In recent years, increasing attention has been paid to PD, thereby improving understanding of PD. In the past 30 years, with the development of medical technology, the overall condition of patients with PD has significantly improved (121). Although studies increasingly involve research on PD, no satisfactory treatment has been found.

Exosomes have been demonstrated to be important in the development and treatment of many diseases. For example, the level of CD8 in exosomes from patients with chronic hepatitis C is higher than that in healthy people, and appears to be associated with inflammatory activity and fibrosis (122); Exogenous mir-145–5p delivered by exosomes can effectively inhibit the development of pancreatic ductal adenocarcinoma (123). As new carriers, exosomes have been used to provide nucleic acids for the treatment of cancer (124). Therefore, exosomes are useful in the treatment of many diseases. Only a few clinical trials have examined exosomes in the treatment of PD; therefore, more studies are needed. Compared with MSC injection therapy, exosomes are safer and easier to control. Exosomes are natural nanoparticle biological carriers with stable properties and good membrane permeability, which can cross the blood-brain barrier (125). Moreover, exosomes can recognize specific cells, thus resulting in better curative effects and fewer off-target effects than other biological carriers (98). Exosomes are easy to obtain and can be separated from the blood, urine, saliva, amniotic fluid, malignant ascites, bronchoalveolar lavage fluid, synovial fluid and breast milk (97). Therefore, exosome-based drug therapy may be a potential treatment for many diseases.

## Conclusion and future prospects

This review highlighted the important roles of MSC-derived exosomes in the treatment of PD. The precise mechanism of MSC-derived exosomes involved in in PD treatment remains unknown but may involve crossing of the blood-brain barrier, intercellular communication, neuroprotection and anti-inflammatory effects. Crossing the blood-brain barrier and intercellular communication may be especially important in PD treatment. The neuroprotective and anti-inflammatory effects still require further research.

Although MSC-derived exosome therapy is a promising modality for PD, several problems require further attention and exploration: (1) MSC-derived exosomes have demonstrated great potential and availability for treating PD in animal models. However, research in large animal models and humans is lacking and remains to be conducted. In addition, randomized controlled trials should be performed to confirm the therapeutic effects of exosomes. (2) Exosomes can be secreted by all cells and have a wide range of sources. What types of cells should be extracted for research? Olfactory ensheathing cells, a special type glial cells, are the best materials for nerve repair and can release exosomes. Therefore, these cells may be a good choice.

## Author contributions

S-fL, J-lZ, and L-yL are responsible for writing the manuscript. M-mL, L-cY, and X-rC are responsible for data collection. C-nC and SL are mainly responsible for reviewing and revising the article. All authors contributed to the article and approved the submitted version.

## Funding

This work was supported by grants from the Natural Science Foundation of Fujian Province of China (Grant number 2019J01164), the Scientific Foundation of Quanzhou City for High Level Talents (Grant number 2019C075R) from C-nC, the Foundation of Science and Technology Bureau of Quanzhou (Grant number 2020CT003) from SL, and the Fujian Provincial Health Technology Project (2019-1-54).

## Conflict of interest

The authors declare that the research was conducted in the absence of any commercial or financial relationships that could be construed as a potential conflict of interest.

## Publisher's note

All claims expressed in this article are solely those of the authors and do not necessarily represent those of their affiliated organizations, or those of the publisher, the editors and the reviewers. Any product that may be evaluated in this article, or claim that may be made by its manufacturer, is not guaranteed or endorsed by the publisher.
